# Why Methylene Blue Is the Only Option for Blocking the cGMP/NO Pathway in The Treatment of Vasoplegic Shock? “Reasons That Reason Itself Does Not Know...”

**DOI:** 10.21470/1678-9741-2021-0957

**Published:** 2021

**Authors:** Paulo Roberto B. Evora, Walter J Gomes

**Affiliations:** 1 Department of Surgery and Anatomy Ribeirão Preto School of Medicine. Faculdade de Medicina de Ribeirão Preto da Universidade de São Paulo (FMRPUSP), Ribeirão Preto, SP, Brazil.; 2 Cardiovascular Surgery Discipline and São Paulo Hospital. Escola Paulista de Medicina. Federal University of São Paulo, São Paulo, Brazil.

There is no reminiscence of the word shock being used in its modern-day form before 1743. However, there is evidence that Hippocrates used the word *exemia* to define a state of being “drained of blood.” Shock or “choc” was first described in a trauma victim in the English translation of Henri-François LeDran’s 1740 text, Traité ou Reflexions Tire’es de la Pratique sur les Playes d’armes à feu (A treatise, or reflections, drawn from practice on gun-shot wounds.) In this text, he describes “choc” as a reaction to the sudden impact. However, the first English writer to use the word shock in its contemporary meaning was James Latta in 1795^[1]^.

George W. Crile, in 1899 suggested in his monograph, “*An Experimental Research into Surgical Shock”* that shock was a state of circulatory collapse due to excessive nervous stimulation. Other competing theories around the turn of the century included one penned by Malcolm in 1905, with the assertion that prolonged vasoconstriction led to the pathophysiological signs and symptoms observed in shock. In the following World War I, research about shock resulted in experiments by Walter B. Cannon of Harvard and William M. Bayliss of London in 1919 showing that an increase in permeability of the capillaries in response to trauma or toxins was responsible for many clinical manifestations of shock. In 1972 Hinshaw and Cox suggested a shock classification system, which is still in use today^[1]^.

Perhaps the best approach to defining the state of shock is that of Robert Hardaway, who begins his discussion by stating, “What shock is not.” This approach is interesting because demonstrates the entire development of circulatory shock as the pathophysiological concepts have changed. Thus, the shock is no longer: 1) Low blood pressure - due to the hypothalamus-pituitary-adrenal axis (release of catecholamines and cortisol) sustaining normal and even supranormal blood pressure; 2) a low pH does not necessarily accompany it - can be normal or even alkalotic depending on the endogenous production of bicarbonate and the compensatory hyperventilation to metabolic acidosis; 3) It is not always accompanied by low cardiac output - hyperdynamic states are associated, for example, with sepsis; 4) It is not due to the exhaustion of the adrenal gland - in pre-death, plasma levels of catecholamines are elevated; 5) Arteriolar vasodilatation does not necessarily coexist - the rule is that vasoconstriction occurs and; 6) There is not necessarily hypovolemia - an example is a cardiogenic shock resulting from acute myocardial infarction. After these considerations, it would remain to define “what shock is,” and, in search of a universal definition, it was concluded that the best definition of the shock state would be: “Inadequate capillary perfusion” or, simply, “Bad tissue perfusion.” These concepts stemming from the 1970s are linked to the Vietnam War.

The discovery and the massive nitric oxide (NO) scientific investment annulled paradigms, starting a new era of knowledge. Vasoplegia associated with systemic inflammatory reaction was lifted to the condition of “enemy to be overcome” The concepts emerging from the observations acquired during the first 15 years of using methylene blue (MB) for treatment of vasoplegic syndrome (VS) during cardiac surgery have established some critical topics. But, we still feel that the cyclic guanosine monophosphate (cGMP) importance is underestimated in the medical literature.

In 2009, we published a personal statement centered on MB as a treatment of VS in cardiac surgery, including fifteen years of questions, answers, doubts, and certainties^[2]^. Some repetitive observations can be applied to VS: (1) MB is safe at the recommended doses (the lethal dose is 40 mg.Kg-1); (2) the use of MB does not cause endothelial dysfunction; (3) the MB effect is manifested in cases of positive NO regulation; (4) MB itself is not a vasoconstrictor, because by blocking the cGMP pathway, it releases the adenosine 3’5’ - cyclic monophosphate (cAMP) pathway, facilitating the vasoconstrictor effect of epinephrine; (5) the MB may act through this mechanism of “crosstalk,” and its use as a medication of the first choice may not be correct; (6) the most used dosage is 2 mg.Kg^-1^ in IV bolus, followed by the same continuous doses infusion because the plasma concentrations markedly decrease in the first 40 minutes. Although there are no definitive randomized controlled trials, the MB used in the treatment of VS following cardiac surgery is currently the best, safest, and cheapest option; (8) however, a possible ‘window of opportunity for the effectiveness of MB has not yet been clearly established for humans.

This editorial report has the primary purpose of performing a simple exercise of logic. It is well established^[3,4]^:

1. The inflammatory reaction is present in all types of circulatory shock.
2. As the vasoplegia extent goes on, a “vasoplegic endothelial dysfunction” with catecholamine-resistant arterial hypotension leads irreversibly to death.
3. This dysfunction is mediated by the cGMP / NO system and is blocked by MB.
4. Currently, the blockade of soluble guanylate cyclase (sGC) by MB, despite the most diverse criticisms, is still a matter of controversy, although this drug was first used in humans over 100 years ago.

What is the basis for the proposed logic exercise?

The NO pathway blocking is already part of the vasoplegia therapeutic arsenal. However, assuming that the MB is at least controversial, why the search for an alternative drug fall short of investment?

Following the original report in 1994, the VS was afterward met with skepticism and even denial^[5]^. However, the VS has steadily been recognized as a common complication in cardiac surgery, occurring in 20% to 27% of patients, and associated with significant morbidity and mortality. Cost analysis showed that in the United States the vasoplegia imposed an increase in ICU length of stay of 166,000 days, coming to at least $1.4 billion in cost annually. Therefore, if prevented, could potentially save in healthcare spending^[6]^.

Restrictions on MB can be disguised as a mandatory objective. From the physiological point of view, a bias toward the blockage of the cGMP/ NO pathway concealed its fundamental importance. In other words, the resistance to MB use can blunt this importance.

Quoting Blaise Pascoal’s should be “Reasons That Reason Itself does not know ...?”, But the science and pharmaceutical industry are in debt.
